# On Construction of Real-Time Monitoring System for Sport Cruiser Motorcycles Using NB-IoT and Multi-Sensors

**DOI:** 10.3390/s24237484

**Published:** 2024-11-23

**Authors:** Endah Kristiani, Tzu-Hao Yu, Chao-Tung Yang

**Affiliations:** 1 Department of Computer Science, Tunghai University, Taichung City 407224, Taiwan; endahkristi@gmail.com (E.K.); tzuhao@thu.edu.tw (T.-H.Y.); 2 Department of Informatics, Krida Wacana Christian University, Jakarta 11470, Indonesia; 3 Research Center for Smart Sustainable Circular Economy, Tunghai University, No. 1727, Sec. 4, Taiwan Boulevard, Taichung City 407224, Taiwan

**Keywords:** multi sensor, IoT, NB-IoT, MQTT, real-time monitoring

## Abstract

This study leverages IoT technology to develop a real-time monitoring system for large motorcycles. We collaborated with professional mechanics to define the required data types and system architecture, ensuring practicality and efficiency. The system integrates the NB-IoT for efficient remote data transmission and uses MQTT for optimized messaging. It also includes advanced database management and intuitive data visualization for enhancing the user experience. For hardware installation, the system follows strict guidelines to avoid damaging the motorcycle’s original structure, comply with Taiwan’s legal standards, and prevent unauthorized modifications. The implementation of this real-time monitoring system is anticipated to significantly reduce safety risks associated with mechanical failures as it continuously monitors inappropriate driving behaviors and detects mechanical abnormalities in real time. The study indicates that the integration of advanced technologies, such as the NB-IoT and multi-sensor systems, can lead to improved driving safety and operational efficiency. Furthermore, the research suggests that the system’s ability to provide instant notifications and alerts through the platforms’ instant messaging can enhance user responsiveness to potential hazards, thereby contributing to a safer riding experience.

## 1. Introduction

Implementing Narrowband IoT (NB-IoT) technology is essential for improving the efficiency and connection of motorbike real-time monitoring systems. The NB-IoT is primarily designed for low-power, wide-area network applications, making it especially appropriate for scenarios that demand reliable connections and effective data transfer [1]. On the other hand, general Internet of Things (IoT) applications cover a broad range of technologies. For the implementation of vehicle monitoring systems, the low power consumption features of the NB-IoT enable the long-term operation of monitoring devices without regular battery replacements [2]. Furthermore, the NB-IoT’s excellent signal penetration capabilities and capacity to leverage pre-existing cellular network infrastructure significantly lower implementation costs and improve data transmission reliability in a variety of settings. This study highlights how crucial the NB-IoT is to maintaining sport cruiser motorcycle data communication stability, especially in high-speed mobile situations.

The integration of the Internet of Things (IoT) and Narrowband IoT (NB-IoT) technologies is revolutionizing various sectors by enhancing connectivity and data management capabilities. As highlighted by Cengiz et al. [3], the IoT and NB-IoT play pivotal roles in overcoming deployment limitations in smart automation and robotics, offering scalable solutions for diverse applications. These technologies facilitate efficient communication networks, as discussed by Sharma et al. [4], where AI and machine learning-driven IoT systems are emerging as intelligent solutions for sensor communication networks. Furthermore, Lin et al. [5] demonstrate the application of the NB-IoT in decision support systems for building information management, showcasing its potential in optimizing resource allocation and operational efficiency. In healthcare, Vanteru et al. [6] explore a multi-sensor monitoring system using LoWPAN-based architecture, underscoring the importance of the IoT in real-time health monitoring and data analysis. Collectively, these studies illustrate the transformative impact of the IoT and NB-IoT across various domains, emphasizing their role in enhancing connectivity, data processing, and decision-making processes.

Recent advancements in motorcycle technology and safety have been significantly influenced by innovative research in various domains, as evidenced by recent scholarly articles. Will et al. [7] explore methodological considerations in naturalistic motorcycle riding investigations, emphasizing the use of gg diagrams for rider profile detection. This approach enhances the understanding of rider behavior and safety by providing a detailed analysis of riding patterns. Meanwhile, Capriglione et al. [8] focus on the development of soft sensors for instrument fault accommodation in semiactive motorcycle suspension systems. These sensors are crucial for maintaining optimal suspension performance, thereby improving ride comfort and safety. In the realm of electric motorcycles, Lee et al. [9] introduce a flexible five-in-one microsensor designed for real-time wireless microscopic diagnosis within fuel cell stack range extenders. This technology facilitates efficient monitoring and maintenance of electric motorcycles, ensuring their reliability and performance. Additionally, Lee et al. [10] present a flexible 4-in-1 microsensor for the in situ diagnosis of electric motorcycle fuel cell range extenders, further contributing to the advancement of diagnostic tools in electric vehicle technology. Collectively, these studies underscore the ongoing efforts to enhance motorcycle safety and performance through technological innovation and methodological rigor.

The purpose of this study is to fill a specific technical gap in the real-time monitoring of sport cruiser bikes, specifically a lack of dependable and effective data transmission techniques needed for mobile settings involving high speeds. The advantages of the NB-IoT, a low-power, wide-area application that offers better signal penetration and connection stability, are not completely realized by existing solutions, which usually use conventional IoT technology. Furthermore, a lot of existing systems do not include multi-sensor technologies that can offer thorough data collecting and analysis. This is essential for the precise tracking of vehicle performance and the identification of mechanical anomalies. In order to address the gap in current motorbike monitoring systems, which have trouble with data reliability and operational efficiency, this study focuses on the integration of the NB-IoT and the MQTT protocol to improve data transmission efficiency and real-time communication. Therefore, the contributions of this study are listed as follows:

1. Development of a real-time monitoring system for sport cruiser motorcycles using NB-IoT technology to enhance driving safety and maintain mechanical failures.
2. Integration of NB-IoT technology and the MQTT protocol for efficient data transmission and real-time data communication.
3. Implement multi-sensor technologies for comprehensive data collection and analysis for big motorcycles.

## 2. Background Review and Related Works

This section performs a comprehensive literature review to explore Internet of Things (IoT) technology and its innovative use in traffic monitoring systems, especially big bike monitoring applications. This includes a detailed exploration of the effectiveness of NB-IoT technology in remote data transmission, as well as the key role of the MQTT protocol in enabling real-time data communication. In addition, the literature review will cover methods for combining multi-sensor technologies, how to effectively collect and analyze operational data, and how database management and data visualization tools can enhance user experience and analysis capabilities.

### 2.1. Internet of Things

The Internet of Things (IoT), as an emerging technology paradigm that connects smart physical devices, aims to provide users with a series of integrated smart services. Sobin et al. [11] discussed in detail the key technologies, application scenarios, and challenges of the Internet of Things in their review. IoT technology enables various smart devices to collect and exchange data, thereby supporting the development of smart homes, smart cities, smart grids, and other application fields. Since Kevin Ashton first proposed the concept of the Internet of Things in 1999, the Internet of Things has gradually transformed from theory into practical applications through the application of technologies such as radio frequency identification (RFID), wireless sensor networks (WSN), and machine-to-machine communication (M2M). However, with the rapid development of IoT technology, challenges such as security and privacy protection, data management and processing, and interoperability between devices have gradually emerged. These challenges have put forward new requirements for the sustainable development and widespread application of the IoT [12].

In research on the Internet of Things related to motorcycles, Tashfia et al. [13] proposed a monitoring solution that combines the Internet of Things (IoT) and expert systems to improve motorcycle riding safety. The solution integrates multiple sensors, including smart helmets, alcohol detection, engine temperature monitoring, vehicle speed and distance monitoring of nearby vehicles, and real-time location tracking. In the event of an accident, the system can immediately send Global Positioning System (GPS) information containing the accident location to the authorized contact via SMS. However, the study did not involve monitoring motorcycle driving behavior and did not provide early warning of possible risks. Especially for rental companies or fleet managers, understanding each driver’s behavior and status is crucial. In addition, based on Taiwan’s legal regulations, installing the device inside a helmet may cause safety and illegal concerns, change the original protective structure of the helmet, and require frequent charging.

The research by KumarJha and Pabla [14] proposed an engine lubricating oil instant monitoring system (EOM) based on Internet of Things (IoT) technology. The system integrates multiple sensing devices, such as light-dependent resistor (LDR) sensors, LM35 temperature sensors, and ultrasonic sensors, to achieve real-time monitoring of lubricating oil quality, temperature, and oil level during engine operation. The system can instantly transmit the collected data from these sensors to the display unit, enabling real-time diagnosis and lubricant status monitoring based on real-time information. Although the theoretical framework of this research is complete, the actual application may require intrusive modifications to the vehicle, as Taiwan’s current regulations prohibit the modification of the electric system and engine. This study did not utilize the vehicle and instead focused solely on its transmission method.

Pradeep et al. [15] proposed an innovative Internet of Things (IoT) technology application and developed a smart motorcycle accident detection system [11]. This system integrates a variety of sensor technologies to realize real-time monitoring and safety management of the vehicle’s driving status. When a collision or other safety incident occurs, the system can automatically send an alert to a preset emergency contact person and has multiple functions, such as fuel theft warning and speed warning. The purpose is to reduce the risk of traffic accidents and enhance riding safety. However, the implementation of this system requires the modification of the safety helmet, there are fewer cases of fuel theft and vehicle overloading in the Taiwanese market, and there are regulatory challenges that may arise from structural changes to the safety helmet.

### 2.2. Low-Power Wide-Area Network

Low-power wide-area network (LPWAN) technology, with its unique advantages in long-distance communication and low-power operation, has become a key driving force in the Internet of Things (IoT) field [16]. LPWAN technology enables devices to communicate over wide geographic ranges while maintaining low energy consumption, thereby extending battery life. This makes LPWAN technology particularly suitable for IoT applications that require long-term operation and are often located in remote or difficult-to-access areas, such as smart agriculture, urban infrastructure monitoring, environmental monitoring, and asset tracking [17].

Among LPWAN’s many technologies, the Narrowband IoT (NB-IoT), Long Range (LoRa), Sigfox, and LTE-M are currently the most widely discussed and deployed technologies. Each technology has its own specific application scenarios and advantages. For example, the NB-IoT has excellent building penetration capabilities and high connection efficiency, making it suitable for applications in smart cities and industrial environments. LoRa and Sigfox are suitable for a wide range of commercial and industrial applications due to their openness and low-cost deployment characteristics [18]).

It is particularly worth mentioning that these LPWAN technologies play a vital role in the development of smart cities. LPWAN technology supports the connection of a large number of low-power devices in smart cities; from smart lighting to traffic monitoring systems to waste management and water quality monitoring, it provides an efficient and sustainable solution for urban management and services [19]).

### 2.3. Narrowband Internet of Things

The Narrowband IoT (NB-IoT) is a cellular-based low-power wide-area network technology (LPWAN) designed to connect numerous IoT devices globally [20]. As part of the 3GPP standards, the NB-IoT provides an optimized solution that can operate under existing cellular network architecture, specifically utilizing existing LTE and GSM networks. The technology is known for its excellent indoor coverage, low cost, long battery life, and high connection density. The NB-IoT supports a wide range of application scenarios, including smart metering, smart cities, asset tracking, environmental monitoring, and agriculture, making it one of the key technologies promoting the development of the Internet of Things.

NB-IoT technology is able to transmit small amounts of data using a very narrow bandwidth (approximately 200 kHz), which is ideal for applications that do not require large amounts of data exchange. In addition, the NB-IoT supports technologies such as eDRX (Extended Idle Cycle) and PSM (Power Saving Mode), which can significantly extend the operating time of a device on a single battery charge, sometimes even for several years. Therefore, the NB-IoT is particularly suitable for scenarios that require long-term operation and are inconvenient to replace batteries [21].

In the study by Marini et al. [22], the potential of the NB-IoT in smart city applications was deeply discussed. Smart city projects usually cover a wide range and require a large number of sensors and devices for data collection and analysis to achieve intelligent and automated city management. The low power consumption characteristics of NB-IoT technology make it ideal for achieving these goals, especially in areas such as traffic monitoring, vehicle management, smart lighting, and waste management. Compared with other LPWAN technologies, such as LoRa and Sigfox, the NB-IoT not only provides better signal penetration capabilities and wider operator support but also can directly utilize existing cellular network infrastructure, thereby reducing deployment costs and enhancing the speed.

In the study by Wang et al. [23], by comparing the performance [23], special emphasis was placed on performance differences of these technologies in aerial application scenarios [16] by comparing the performance of four popular low-power wide-area network (LPWAN) technologies, the NB-IoT, LTE Cat-M1, Sigfox, and LoRa, in high-speed mobile conditions, special emphasis was placed on Performance differences of these technologies in aerial application scenarios [16]. conditions. The experiment was conducted using a drone flying along a 10-km highway at a speed of 70 km per hour as a mobile IoT terminal device, aiming to simulate communication conditions under high-speed movement. Among the four technologies, the NB-IoT performs well with its advantages based on cellular networks, especially in terms of stability and signal coverage. NB-IoT technology is designed to leverage existing LTE network infrastructure to provide efficient communications within narrow frequency bands. This design enables the NB-IoT to have excellent penetration capabilities in indoor and underground environments and is also expected to maintain stable connection performance under high-speed mobile conditions. However, since the NB-IoT is mainly optimized for static or low-speed moving scenarios, when objects move at higher speeds, such as the drone scenario used in this study, its performance may be affected, especially in maintaining connections and Data transmission stability.

### 2.4. Related Works

Hanif et al. [24] developed a motorcycle pothole detection system aimed at improving road driving safety through proximity sensors [17]. The team implemented the research in three phases: developing a pothole road identification system, designing the system, and conducting testing. The core technology is based on a ToF (Time of Flight) proximity sensor, combined with a gyroscope and Hall effect sensor, and performs data processing through an Arduino microcontroller to identify the road conditions ahead and provide real-time warning to the driver.

Hidayanti et al. [25] designed a motorcycle security system using a fingerprint sensor and Arduino Uno microcontroller [18]. By using a fingerprint sensor to start the motorcycle instead of the traditional ignition key, this system enhances the anti-theft function and improves motorcycle safety. The system design includes a dual start key as a steering wheel lock and a fingerprint as vehicle access. Research shows that the user’s fingerprint can successfully access the motorcycle 95% of the time. The security system uses a two-channel relay module as the connection between hardware and software, a fingerprint module to capture and compare fingerprint patterns, and an Arduino Uno as a tool for response reception and command execution.

Artono et al. [26] developed a motorcycle safety system that combines SMS alerts and GPS tracking functions to improve the safety of motorcycles parked in public places [19]. This system uses the SIM808 GSM module to send alarm information and provides latitude and longitude coordinates via a GPS tracker to track the stolen motorcycle’s location using Google Maps. The system passed the test and can monitor coordinate changes through Google Maps and display the movement trajectory of the motorcycle when integrating the motorcycle system.

Bante et al. [27] explored an exhaust gas sensing system based on sensors to measure motorcycle emissions in real time [20]. This study, based on the background of widespread use of motorcycles in developing countries, designed and established a low-cost, real-time exhaust monitoring system using MQ-2, MQ-7, MQ-135, and MG-811 sensors to detect nitric oxide (NO), carbon monoxide (CO), and hydrocarbon (HC) emissions from general motorcycles. Tests were conducted at the Energy Conversion Laboratory at the G. H. Raisoni School of Engineering to evaluate the sensor-based emissions device’s performance under a variety of conditions, including load, speed, and biodiesel usage. It was found that the average errors of CO, NOx, and HC were 6.96%, 7.89%, and 8.04%, respectively.

Salman et al. [28] demonstrated the practical utility of MQTT in IoT applications by implementing a smart home monitoring system based on the MQTT protocol [21]. The system uses the MQTT protocol to collect, transmit, and monitor temperature and humidity data, which not only demonstrates the advantages of MQTT in data transmission efficiency but also proves its important role in maintaining system reliability. The successful implementation of this system further proves the strong potential and wide applicability of MQTT as an IoT communication protocol, especially in smart applications that require efficient and reliable communication.

In their research, Kegenbekov (2022) et al. [29] investigated the application of the MQTT protocol to transmit vehicle telemetry data in the field of transportation logistics. The research focuses on establishing a hierarchical structure that collects vehicle operating parameters such as geographical location, speed, and engine temperature to optimize logistics operations. The implementation of the MQTT protocol demonstrates its advantages in enhancing data transmission efficiency and system reliability. In addition, the research highlights the applicability and potential of MQTT in the field of IoT communication, providing an effective solution for improving the efficiency of logistics transportation and real-time monitoring of vehicle status. This research not only highlights the role of MQTT in promoting the development of the logistics and transportation industry but also opens up new possibilities for future technological innovation and application.

## 3. Research Methodology and Framework

In this section, the research framework of the study is introduced, which includes a detailed description of the architecture.

### 3.1. System Architecture

**Figure 1:**
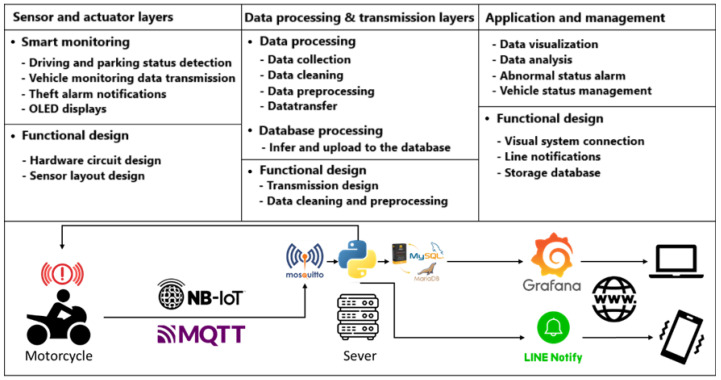
Layers’ functions and design.

Aiming at the operational challenges of sport cruiser motorcycles, this study develops a comprehensive and sophisticated monitoring system. The design of this system integrates three key layers: the sensor and actuator layer, the data processing and transmission layer, and the application and management layer to form a closely connected and efficient operating architecture, as shown in Figure 1.

At the sensor and actuator layer, the system focuses on capturing real-time operating data using a variety of sensors, such as GPS modules, vibration sensors, voltage sensors, and other vehicle status monitoring sensors, to conduct comprehensive data collection. The integration of these sensors and microprocessors not only provides an accurate source of data but also lays a solid foundation for subsequent data analysis. Data processing is handled by the data processing and transmission layer, which uses Python for data cleaning, outlier detection, and conversion. This layer, when combined with NB-IoT technology and the MQTT protocol, not only achieves efficient data transmission but also ensures smooth data communication between different modules. Additionally, this level of computing power enables more detailed data analysis, sending the processing results back to the sensor and actuator layers for real-time response.

Ultimately, applications and management focus on transforming complex data into practical applications and management strategies. Grafana, a visualization tool, clearly displays data and provides decision-makers with easily understandable information. This level also integrates communication channels, such as LINE Notify, to provide users with instant notifications and alerts. This not only enhances the user experience but also provides customized management solutions for leasing operators, fleet managers, and individual users. In summary, this research’s monitoring system, with its multi-layered, high-efficiency, and low-power architecture, not only enhances the safety and efficiency of vehicle operation but also demonstrates excellent adaptability and scalability. The system can meet a wide range of application requirements and provides comprehensive and in-depth technical support for the operation management of big bikes.

We developed a low-power monitoring system for the operation of sport cruiser motorcycles during the experimental design process. We select and configure the system development environment to ensure technical stability and reliability. The core development environment uses macOS 13.5.2 as the operating system platform, combined with Arduino IDE version 1.8.19 to develop the ESP-32 microcontroller. The microcontroller uses version 2.0.9 of the SDK to ensure the best compatibility with the hardware. Capacity. We chose the Python 3.11 version for back-end services and data processing, fully utilizing its extensive standard library and third-party function libraries.

**Figure 2:**
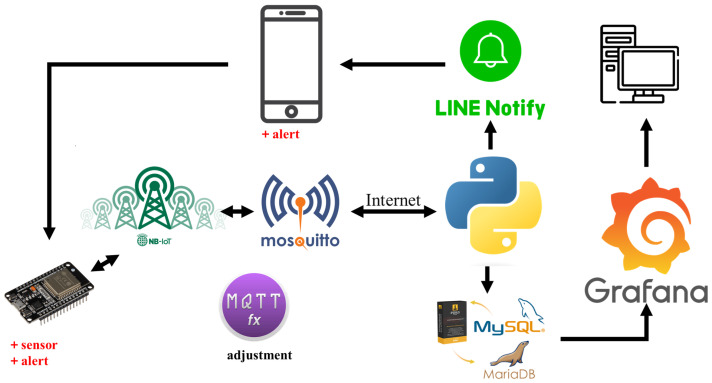
System architecture.

To improve system adjustment efficiency, we introduced MQTT.FX as a key client tool of the MQTT protocol. The use of MQTT.FX mainly focuses on the system adjustment phase, providing an intuitive interface to monitor and analyze MQTT message flows in real time. Its simple and effective functional design can quickly identify communication problems and make appropriate adjustments during development, thereby enhancing the system’s data communication reliability and accuracy. This configuration lays a solid foundation for efficient development and future maintenance of the system. Figure 2 illustrates the overall architecture, elucidating each component in detail in the following sections.

Integrating a cloud computing system into the existing architecture of the real-time monitoring system for sport cruiser motorcycles could significantly improve its capabilities [30]. The current system architecture consists of three key layers: the sensor and actuator layer, the data processing and transmission layer, and the application and management layer. By adding a cloud computing layer, we can improve data storage, processing power, and scalability, which are essential for handling the increasing volume of data generated by multiple sensors. Incorporating cloud computing would allow for centralized data management, enabling real-time data analysis and storage in a more efficient manner. This would facilitate the use of advanced analytics and machine learning algorithms to predict maintenance needs and detect anomalies, thus enhancing the system’s reliability and performance. However, managing large amounts of data generated by multiple agents can be challenging in cloud computing. Therefore, a paper by Ren et al. [31] investigates the distributed group asynchronous consensus problem for cyber-physical systems (CPSs) characterized by unknown dynamics and switching topologies. Their paper outlines the design of a distributed asynchronous switching observer that enables agents to accurately track the leader’s state information despite potential delays in topology switching. The findings highlight the robustness of the proposed approach in maintaining stability and performance in dynamic environments.

Furthermore, cloud services could provide a user-friendly interface for data visualization, similar to the current use of Grafana, but with added capabilities for remote access and collaboration among users. Furthermore, the cloud architecture could support the integration of various data sources, allowing a more comprehensive analysis of vehicle performance and environmental conditions. This would enable managers and individual users to make informed decisions based on a holistic view of the data collected from motorcycles. In general, the addition of a cloud computing system would not only optimize the existing architecture but also pave the way for future enhancements in vehicle monitoring and management.

### 3.2. Data Transmission

#### 3.2.1. Wireless Network Transmission

To achieve effective monitoring of big motorcycles, the system designed in this study involves key technology choices, especially in the decision-making of data transmission technology. We initially evaluated Personal Area Networks (PANs) and local area networks (LANs) as transmission options, but their transmission distance limitations made these technologies unsuitable for this system’s needs. PAN and LAN are mainly suitable for stable connections in a smaller range, but for big bikes that need to operate in a wider area, the limitations of these technologies are obvious. The analysis then turned to wide area networks (WANs) and low-power wide area networks (LPWANs). Although WAN has wider coverage, LPWAN shows more significant advantages in terms of energy efficiency and cost. The low power consumption characteristics of LPWAN, combined with its stable long-distance communication capabilities, make it an ideal choice, especially for applications that require long-term operation and low maintenance costs. Therefore, we ultimately decided to use LPWAN as the main data transmission technology, taking into account its advantages. This decision not only ensures the monitoring system’s practicality and reliability but also provides a solid technical foundation for extensive and continuous monitoring of big motorcycles.

#### 3.2.2. LPWAN

The study compares the characteristics and limitations of LoRa, Sigfox, and the NB-IoT when choosing LPWAN technology for big motorcycles’ real-time monitoring systems. LoRa is widely adopted due to its long-distance communication and simple network structure. However, its capacity is limited by downlink transmission and idle time after transmission, which may affect its feasibility. Sigfox is a proprietary Ultra Narrowband solution with a data transmission rate of 100 bps and a maximum payload of 12 bytes in a single packet. It has limitations such as not using collision detection/avoidance technologies, duty cycle limitations, power spectral density radiation limitations, strong interference with adjacent broadband systems, and no data confirmation mechanism. The NB-IoT, a 3GPP-standard LPWAN wireless communication standard, has high compatibility with 4G LTE networks, superior network coverage, data transmission stability, and cost-effectiveness. Field tests show that the NB-IoT can maintain stable and reliable data transmission on high-speed roads, making it the main data transmission mechanism for monitoring big bikes.

#### 3.2.3. Transport Protocol and Proxy Server

This study selected the MQTT (Message Queuing Telemetry Transport) protocol and Mosquitto as the message proxy server to facilitate data communication within the real-time monitoring system of the sport cruiser motorcycle. MQTT is a lightweight messaging protocol based on the publish/subscribe model, designed to cope with low bandwidth and unstable network environments. Its core components include publishers, subscribers, and MQTT proxy servers. Publishers are responsible for sending messages to specific topics, while subscribers subscribe to these topics to receive emails. The proxy server is responsible for receiving messages from publishers and distributing them to subscribers without direct interaction between publishers and subscribers. The MQTT library implements MQTT by providing an interface between clients and proxy servers. The publication of an MQTT message contains a payload, which is custom data sent by the publisher to the proxy server. These messages are diverse and can contain a variety of data types, from sensor readings and commands to important status updates. In this study, the messages sent by MQTT are transmitted in JSON format, providing flexible and structured data expression.

Mosquitto is an open-source (EPL/EDL-licensed) message broker that implements multiple versions of the MQTT protocol, including 5.0, 3.1.1, and 3.1. It is lightweight and suitable for everything from low-power single-board computers to full servers. Mosquitto also provides a C language library for implementing MQTT clients as well as the popular mosquitto_pub and mosquitto_sub command-line MQTT clients. Mosquitto’s high portability allows it to be used on a variety of platforms. To facilitate testing, the Mosquitto project runs a test server to test clients under different environments, including TLS encryption and WebSockets. Overall, the combination of MQTT and Mosquitto provides a reliable and efficient data transmission solution that is suitable for this study’s requirements for low power consumption, high efficiency, and flexibility. This configuration ensures that the system can maintain efficient operation even when the network connection is unstable while providing flexible support for different data formats.

### 3.3. Data Cleaning and Storage

Python plays a key role, providing comprehensive solutions for data collection, processing, analysis, and storage. To achieve real-time sensor data collection, Python uses the paho-mqtt library to exchange data with the Mosquitto agent. Python processes these data, uploads them to the MySQL database via the MySQL Connector library, and then cleans and filters them to meet analysis and storage requirements. The data cleaning process includes the following steps:

- First, we verify the completeness and correctness of the data received from the sensor. To check the data format, we use regular expressions (Regex) to ensure that each data point meets the expected format requirements.
- Missing value processing: During the data transmission process, missing values sometimes occur. To ensure data continuity, we will use interpolation or mean imputation to handle the missing values.
- We identified and processed outliers. We use 95% and 5% methods to detect and remove extreme values to ensure the authenticity and reliability of the data.
- Data conversion: Perform the necessary data conversion and formatting according to analysis needs, such as converting timestamps to a standard time format or converting geographical coordinates to a specified coordinate system.
- Data storage: We will store the cleaned data in the MySQL database for further analysis and processing. We designed the database with efficiency and scalability in mind, ensuring quick retrieval and processing of large amounts of data.
- Python plays an important role in the alert mechanism. To achieve real-time alerts, it communicates with the LINE Notify service interface via HTTP requests. When sensor data exceed a preset threshold, Python triggers an alarm to notify the user or manager. Simultaneously, Python performs vehicle status judgment and mode conversion, then relays the results back to the ESP-32 microcontroller for further adjustments.
- PHPAdmin manages data in the MySQL database and offers an intuitive user interface for data query and management. Additionally, Grafana connects in series with the system to provide a visual display of data, facilitating analysis and report generation. Overall, the integration of Python and PHPAdmin not only improves data collection efficiency and storage security but also optimizes the overall system operating effectiveness to meet the needs of this study for a real-time monitoring system.

### 3.4. Data Visualization and Alerting

We selected Grafana as the key tool for data visualization in this study’s sport cruiser motorcycle real-time monitoring system. Grafana has a highly customizable interface and is compatible with a variety of data sources, including MySQL databases. This allows it to obtain real-time data directly from the database and display the vehicle’s operating status through line charts, dashboards, maps, and other methods. In addition, Grafana’s dashboard displays key performance indicators such as temperature, humidity, and speed, while a map interface provides the vehicle’s geographic location and driving route. The system integrates LINE Notify as a multi-dimensional alert system, offering customized notifications to both individual users and fleet managers. It optimizes vehicle operation and anti-theft strategies for individual users, sending alerts in the event of abnormal inclination or suspected theft. It provides real-time monitoring of driving behavior, such as alerts for speeding, sudden acceleration, or sudden deceleration, which helps with risk assessment and management for fleets and leasing operators. These alerts also serve as a data-driven tool for drivers to self-assess and improve their driving skills. Overall, the combination of Grafana and LINE Notify not only improves data visualization and user experience but also strengthens the system’s real-time monitoring and alarm functions, providing comprehensive technical support for the safe management and operation of sport cruiser motorcycles.

### 3.5. Vehicle Status Recognition System

**Figure 3:**
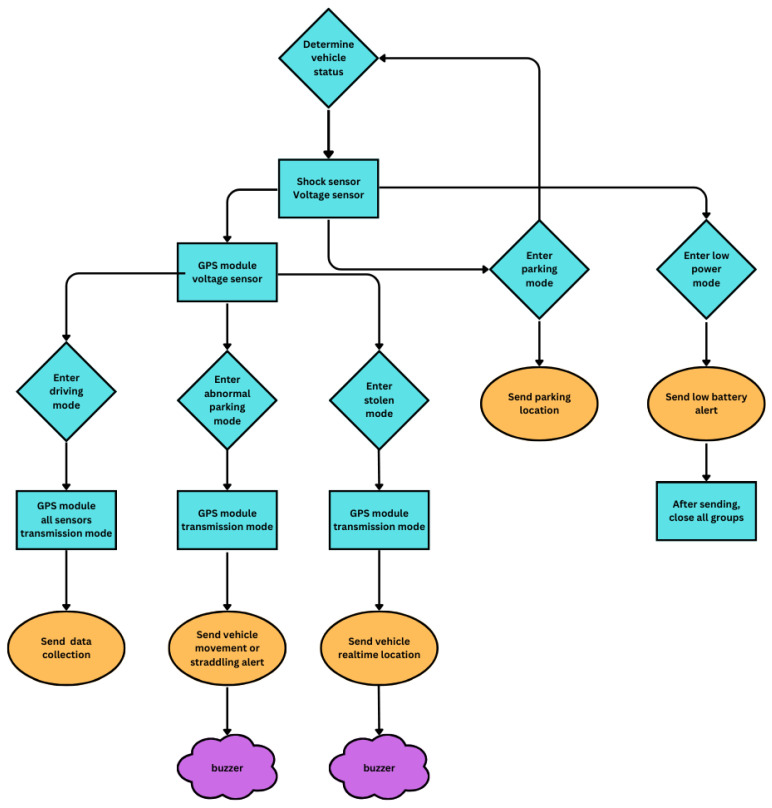
Vehicle status flowchart.

The main challenge was to develop a low-power and automated method for accurately identifying the vehicle’s operating status. Although multiple solutions were initially considered, including using physical buttons or Bluetooth applications for state switching, these methods required additional actions from the user and were inconsistent with the goal of achieving a fully automated system. Furthermore, given that the vehicle battery serves as the system’s primary energy source and has limited power, this solution fails to meet the low-power design requirements for GPS modules with high energy consumption. In addition, GPS modules may encounter poor signal problems in certain parking environments, so relying on GPS to create electronic fences is not feasible. Further research and power consumption comparisons reveal that the vibration sensing module has significant advantages in terms of power consumption and reliability. Subsequent experimental data further revealed a key finding: monitoring battery voltage effectively identifies the vehicle’s operating status. When the engine is Upon stopping the engine, the vehicle maintains the battery voltage at approximately 11 V to 12 V. Upon starting the vehicle, the generator kicks in to recharge the battery and meet the operational power demand, resulting in an increase in battery voltage to Subsequent experiments confirmed this phenomenon. Based on these findings, this study designed multiple vehicle operating modes, such as parking (and parking and moving) mode, driving mode, theft mode, and a low-power mode. The low-power mode activates when the voltage drops below 10 V, ensuring smooth vehicle starting and operation, as illustrated in Figure 3. These modes make full use of changes in battery voltage and vibration as key indicators to identify vehicle status, achieving efficient and automated system operation while also meeting design requirements for low power consumption, effectively improving the overall reliability and practicality of the system.

The following is a detailed explanation of the judgment criteria and functions of each mode:

Parking mode:
  - When the vibration sensor does not detect any vibration, the system will determine that the vehicle is in a parking position. This mode is suitable for normal parking situations.
  - If the vehicle changes from driving mode to parking mode, the system will automatically send the vehicle’s parking position information.
Low battery mode:
  - The system enters low power mode when the voltage sensor measures less than 10 V.
  - After sending the low battery alarm, the microprocessor enters a deep sleep state and disables all sensors to save power.
Driving mode:
  - When the vibration sensor senses vibration and the voltage rises to more than 13 V within 5 min, the system will wake up the GPS module.
  - When the system detects that the speed exceeds 10 KM/H, it will switch to driving mode, activate all sensors to record driving data, and upload and record the data in JSON format through the NB-IoT and MQTT.
Abnormal parking mode:
  - When the vibration sensor detects vibration, but the voltage does not reach 13 V within 5 min, the system will wake up the GPS module.
  - If the system detects no speed exceeding 10 KM/H, it will transition to the parking movement mode. This state could indicate a collision or movement of the vehicle during a parking period. At this point, the system sends an alert and activates the buzzer on the vehicle.
Stolen mode:
  - If the vibration sensor detects vibration and the voltage does not reach 13 V within 5 min, and the vehicle speed exceeds 10 KM/H, the system will switch to theft mode.
  - Considering that modern large-scale heavy-duty vehicles mostly use chip keys, the possibility of illegally starting the vehicle is low. Therefore, this mode primarily involves direct theft of the vehicle during transportation. In this mode, the system will continuously track the vehicle’s position and activate the buzzer.

By combining the above modes, the system can automatically adjust the sensor’s working status according to various situations, achieving accurate vehicle identification and management while effectively saving power.

### 3.6. Energy Saving System

We are focusing on comprehensive vehicle operating status monitoring through a set of diversified sensor strategies. We carefully designed the data transmission and collection mechanism to ensure optimal energy efficiency by only activating the sensors when necessary, thereby effectively reducing the overall energy consumption of the system. The triggers to activate the alarm are described in Table 1.

The system’s sensor configuration covers infrared sensors, six-axis inertial measurement units, global positioning system modules, Narrowband IoT modules, and ambient temperature and humidity sensors. We design each sensor to monitor specific vehicle operating parameters, providing precise and critical data while minimizing energy consumption. Specifically, the six-axis inertial measurement unit tracks the roll angle, pitch angle, and acceleration of the vehicles and detects the occurrence of an abnormal threshold. Trigger the alarm system. The GPS module provides the vehicle’s position, speed, and altitude data to assist in accurate tracking and positioning. We selected NB-IoT modules for data transmission to guarantee the efficiency and stability of data communication. Ambient temperature and humidity sensors evaluate the impact of the surrounding environment on vehicle performance, which is critical for predicting potential engine problems.

**Figure 4:**
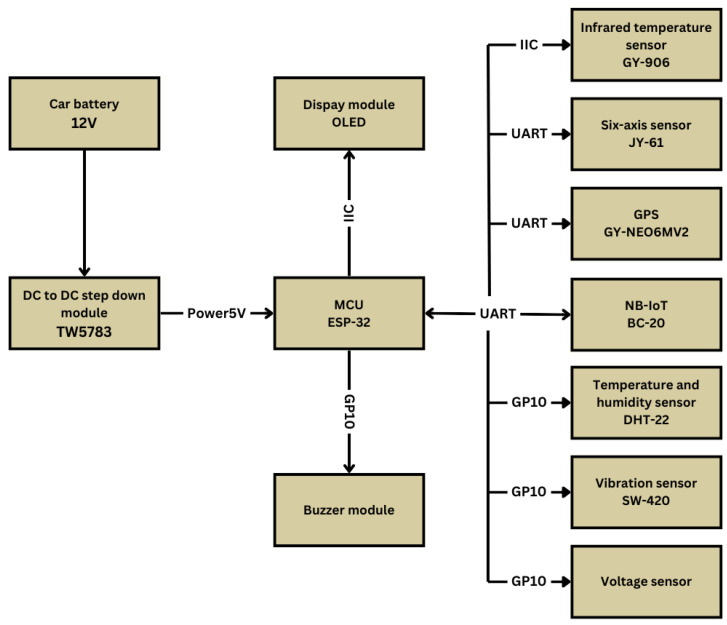
Sensor connection architecture.

Overall, this study’s developed monitoring system not only comprehensively monitors the operating status of big motorcycles through its comprehensive sensor combination and efficient data management strategy but also successfully reduces the system’s overall energy consumption, resulting in energy savings. The goal of efficient monitoring is to provide strong technical support for sport cruiser motorcycle operational safety and efficiency. Figure 4 displays the sensor connection architecture.

#### 3.6.1. Infrared Temperature Sensor

**Figure 5:**
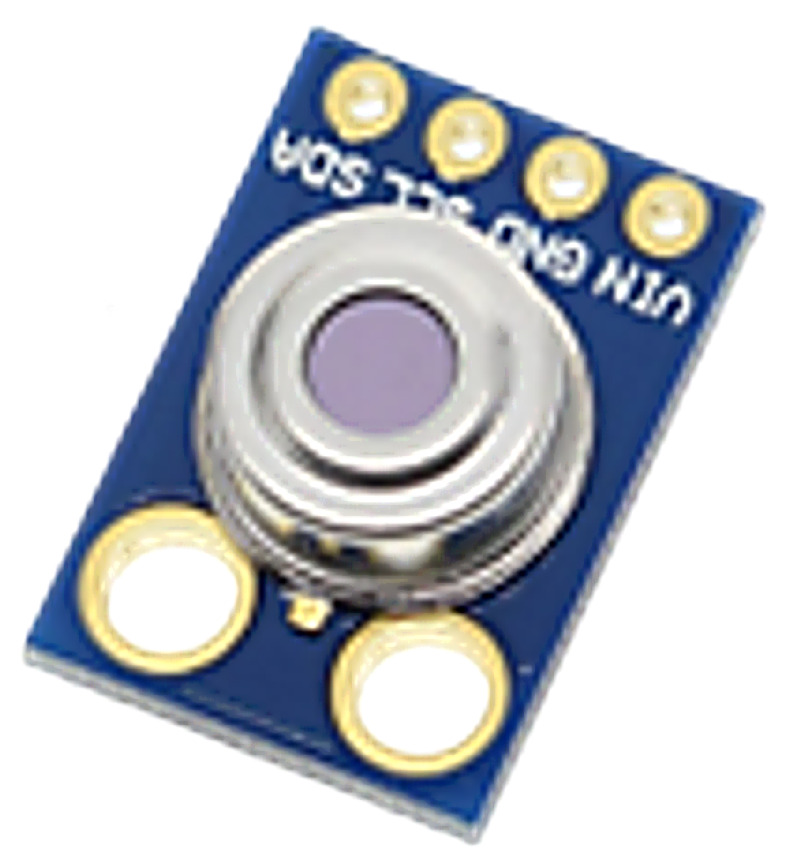
Infrared temperature sensor.

Temperature monitoring is a key parameter in the field of vehicle operation, maintenance management, and real-time monitoring. Especially under high-load or high-performance operating conditions, drastic changes in engine temperature have a significant impact on operating efficiency and fuel consumption. Therefore, this study uses the water tank head temperature as a representative measurement parameter of the engine temperature, aiming to provide a cost-effective monitoring method. Additionally, the study will monitor the surrounding ambient temperature to assess the thermal impact generated by the engine. Table 2 and Figure 5 show the specifications of the infrared temperature sensor.

#### 3.6.2. OLED Display

**Figure 6:**
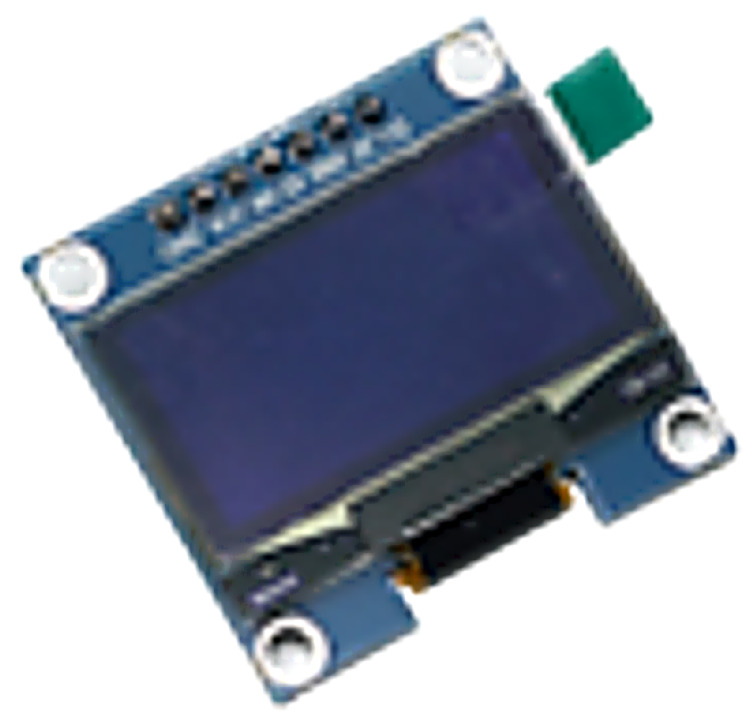
OLED screen.

The system integrates a 1.3-inch OLED display to enhance user interaction efficiency and real-time feedback capabilities. The main function of the display is not only to show whether the system successfully establishes a connection with the back-end service but also to update and display key driving status data in real time, such as vehicle speed, ambient temperature, and GPS positioning information. Table 3 and Figure 6 describes the specifications of the OLED screen.

#### 3.6.3. Six-Axis Sensor

**Figure 7:**
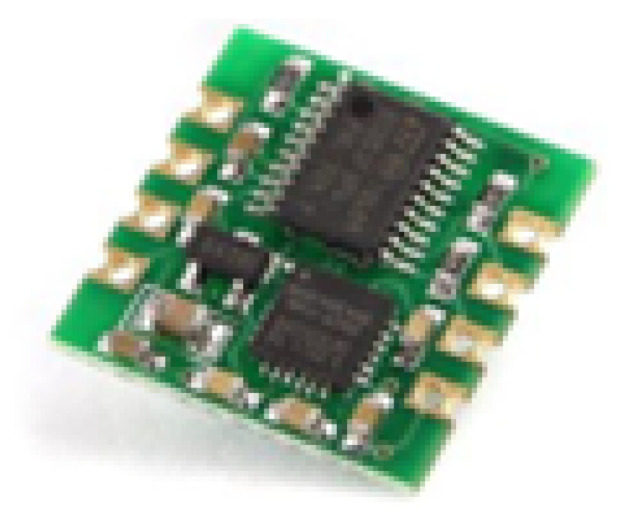
Six-axis accelerometer sensor.

The JY61 module is a highly integrated sensor device that combines gyroscope and accelerometer functions. This module uses a high-performance microprocessor and applies the Kalman dynamic filtering algorithm to achieve the real-time calculation of motion postures. The module can effectively reduce the noise generated during the measurement process thanks to advanced digital filtering technology, thereby improving measurement accuracy. In a dynamic environment, this module can accurately output the current attitude data, and its measurement accuracy can reach 0.2 degrees, demonstrating excellent stability. Table 4 and Figure 7 illustrates the specifications of six-axis accelerometer.

#### 3.6.4. GPS Module

**Figure 8:**
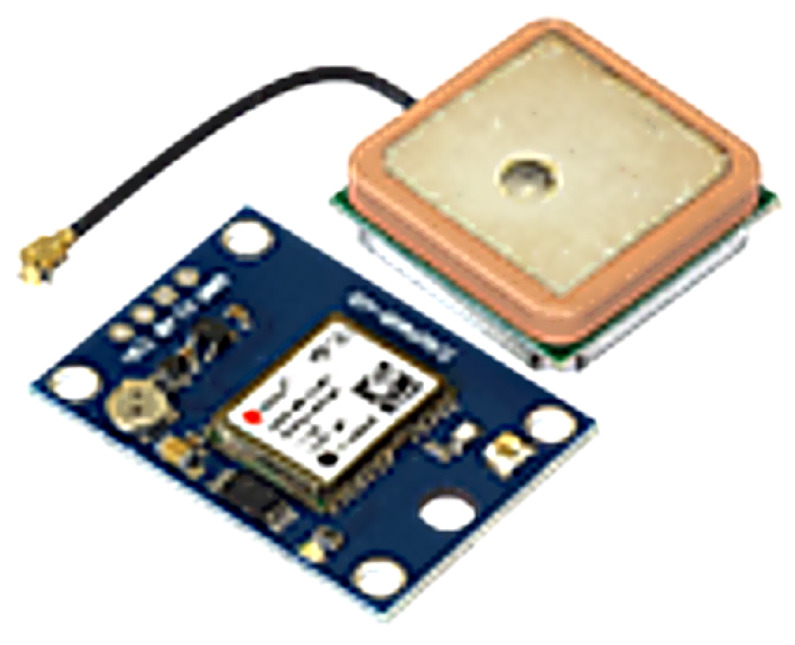
GPS sensor.

We adopted a data fusion strategy to optimize vehicle motion status monitoring by combining data from the Global Positioning System (GPS) and the JY-61 Inertial Measurement Unit (IMU). The GPS system’s longitude and latitude, average altitude, and average speed data, combined with the acceleration, angular velocity, and attitude angle information provided by the JY-61 IMU, create a multi-dimensional motion status monitoring model. Table 5 and Figure 8 shows the specifications of GPS sensor.

#### 3.6.5. NB-IoT Communication Module

**Figure 9:**
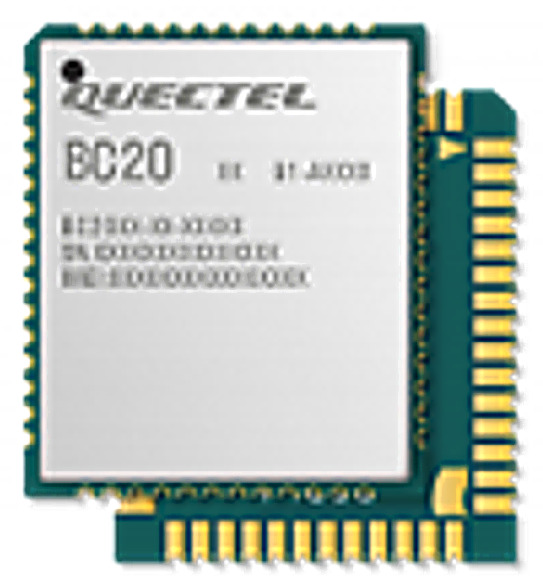
NB-IoT module.

We selected the NB-IoT as the core channel for data transmission, and we used the Message Queuing Telemetry Transport (MQTT) protocol to perform efficient data exchange in JavaScript Object Notation (JSON) format. Despite previous studies raising concerns about data loss in the NB-IoT under high-speed mobile conditions, this study found no such issues during actual testing, indicating that the NB-IoT demonstrates sufficient stability in the project’s application scenarios. and reliability. Table 6 and Figure 9 describe the specifications of the NB-IoT module.

#### 3.6.6. Temperature and Humidity Sensor

**Figure 10:**
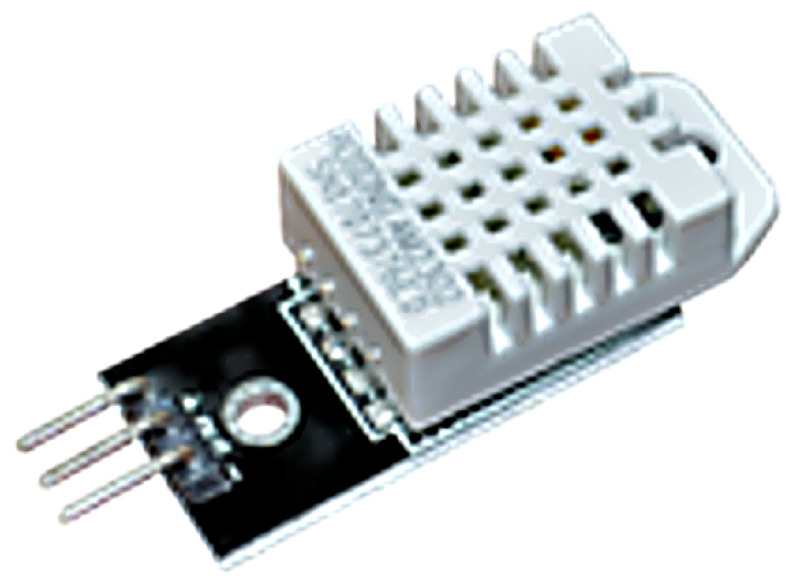
Temperature and humidity sensor.

We introduced a temperature and humidity sensor as a tool to evaluate the impact of the environment on the vehicle engine’s temperature. This move aims to deeply explore the specific impact of ambient temperature and humidity on engine operating performance and further build a comprehensive and sophisticated temperature management strategy. By continuously monitoring and recording ambient temperature and humidity data at different times and dates, this study provides a stable and reliable comparison benchmark for the results obtained from the experiment. Table 7 and Figure 10 illustrates the specifications of temperature and humidity sensor.

#### 3.6.7. Vibration Sensor

**Figure 11:**
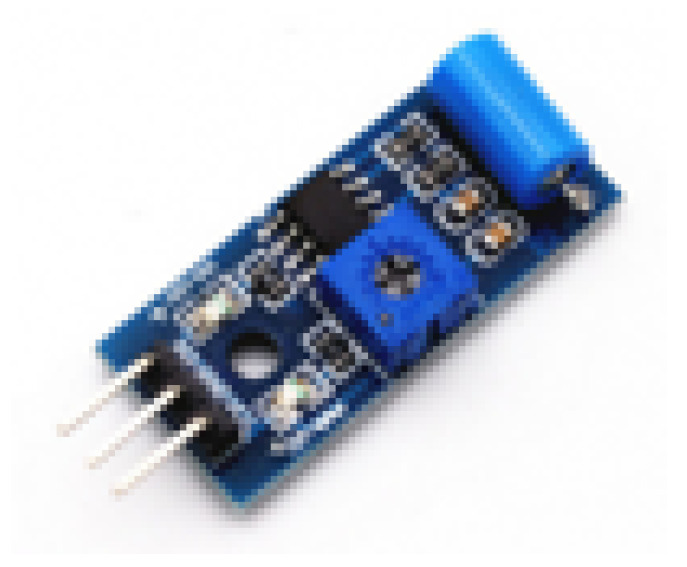
Vibration sensor.

To address the dynamic monitoring issue of the parked motocycles, we have chosen a low-power vibration sensor as the primary monitoring component. This type of vibration sensor’s main feature is that it can efficiently detect any vibration situation. The microcontrol unit (MCU) will automatically activate upon detecting a vibration, either to perform a further status assessment or to issue an alarm. This setting’s main advantage is its fine control of energy management; in a static state without vibration, the vibration sensor consumes very little power and only wakes up the MCU when it detects vibration. This not only extends the system’s operating time but also increases the speed at which it responds to abnormal activities. This way, the system can effectively save power without sacrificing monitoring performance, which is especially suitable for long-term parking monitoring situations. Furthermore, the monitoring solution based on vibration sensors provides a flexible and efficient strategy for motorcycle safety. For instance, the system can promptly conduct a status inspection and safety assessment when the vehicle moves illegally or experiences slight vibrations from natural environmental factors like strong winds. Table 8 and Figure 11 illustrates the specifications of the vibration sensor.

## 4. Experimental Results

### 4.1. System Installation

**Figure 12:**
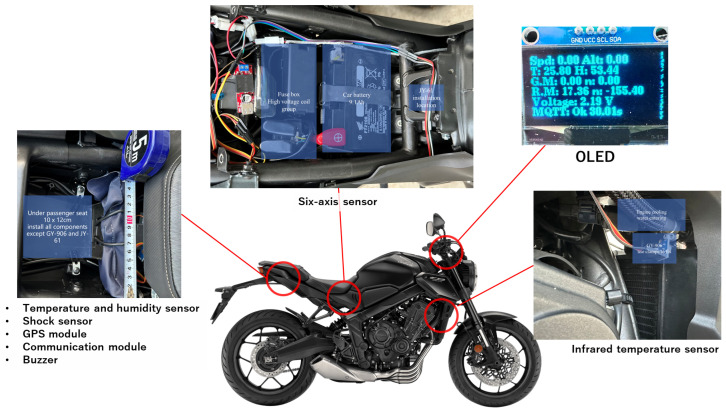
System installation.

In this experiment, we selected the 2023 Honda CB650R as the experimental vehicle to evaluate the performance of the developed system in practical applications. The car retains its original factory configuration, with no recorded abnormalities or damage prior to the test. An official agent introduced the car, appropriately adjusting it to Taiwan’s environmental conditions. The vehicle had a mileage of 9000 km before the start of the experiment, which means it has completed the officially recommended running-in period and has received two maintenance services specified by the original manufacturer to ensure that it is in optimal condition. The system power supply directly connects to the vehicle battery to meet the data collection and analysis requirements of this study, and a DC-DC step-down module adjusts the voltage to 5 volts to meet the system’s operating voltage requirements. We designed this configuration to guarantee the system’s stable operation and power supply reliability while simultaneously reducing energy consumption and enhancing the system’s overall efficiency. The decision to choose the Honda CB650R as the research object was based on its broad market acceptance and representativeness, as well as its excellent vehicle performance and reliability. Additionally, we have optimized and adjusted the vehicle for the unique environmental conditions in Taiwan, ensuring it aligns better with the various performance indicators under evaluation in this study. Through system testing and data analysis of this vehicle model under controlled conditions, this study aims to provide an empirical basis for the performance of the system under actual operating conditions. The implementation part of this research integrates a series of hardware components, which mainly include a main control hardware unit, a JY-61 six-axis sensing module, a GY-906 infrared temperature sensor, and an OLED display unit. We rigorously evaluate and optimize the installation location of each hardware component to achieve specific performance indicators and functional requirements. The main control hardware unit covers ESP-32, the GPS, the NB-IoT, vibration sensing, voltage measurement, buck module, and temperature and humidity sensing module. We accurately place it under the passenger seat, creating an enclosed space measuring 10 cm by 12 cm (Figure 12). The location offers ample space and excellent ventilation, and its quick opening with the vehicle key not only streamlines daily hardware maintenance but also offers convenience for program updates. We install anti-vibration pads and acrylic fixing plates to ensure stable operation of the hardware, thereby reducing damage or impact from bumpy drives.

### 4.2. Visualization and Observation Findings

**Figure 13:**
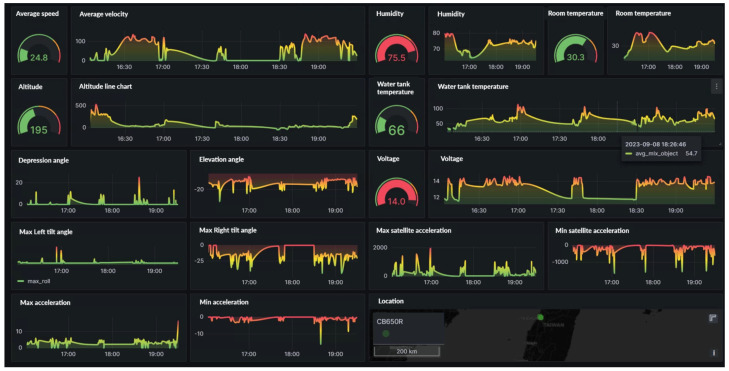
Grafana Interface.

The implementation of this research resulted in the integration with the Grafana system. This integration allows for the real-time display of the vehicle’s operating status, location, speed, and other data, thereby enhancing fleet operation efficiency and management quality. Figure 13 illustrates the interface of Grafana.

**Figure 14:**
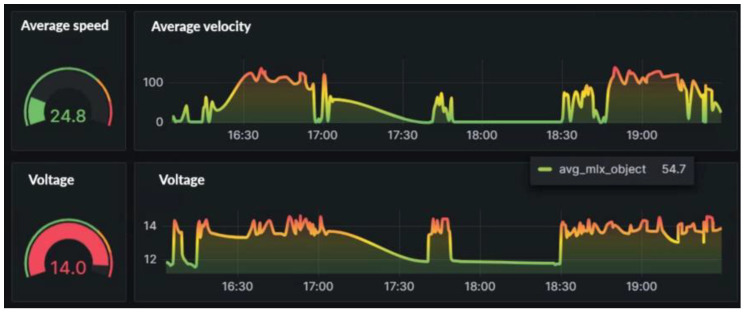
Speed and Voltage.

The analysis in the figure below reveals a significant correlation between speed and voltage. This correlation demonstrates that voltage changes serve as a dependable indicator of the vehicle’s operation. Figure 14 shows the speed and voltage graphs.

**Figure 15:**
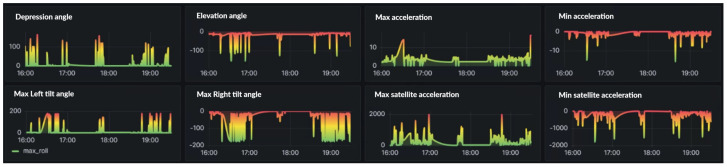
Angle, inclination angle, acceleration, and GPS status.

Next, we can use the three-axis sensor module to obtain key data such as the vehicle’s pitch angle, left and right inclination angle, and acceleration. These data are used to create a visual analysis of driving status. By analyzing the data’s peaks, we can effectively identify and judge improper driving behaviors or improve driving styles. Furthermore, the system includes a GPS module to accurately provide key data, such as acceleration, in a variety of environments. These collected data not only support instant behavioral judgments but also provide a rich data basis for future research directions, further expanding the scope and depth of research applications, such as the relationship with hardware wear or the impact of vehicle status. Figure 15 describes the angle, inclination angle, acceleration, and GPS status.

**Figure 16:**
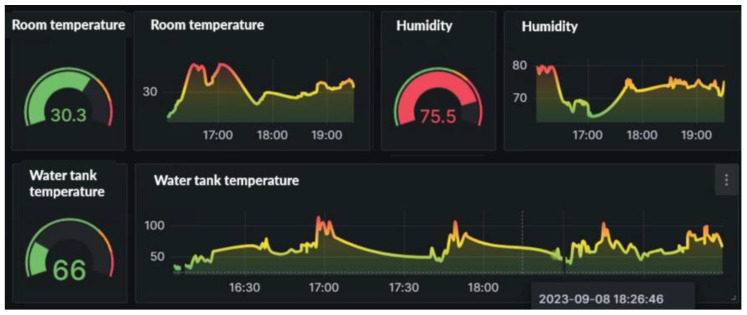
Temperature, humidity, and water tank (engine) temperature status.

In the visual charts for this study, we also included data displays of temperature, humidity, and water tank (engine) temperature. This allows users to intuitively grasp the temperature and humidity of the current environment, as well as changes in water tank temperature, thereby providing more comprehensive vehicle status monitoring. Figure 16 displays the temperature, humidity, and water tank (engine) temperature.

**Figure 17:**
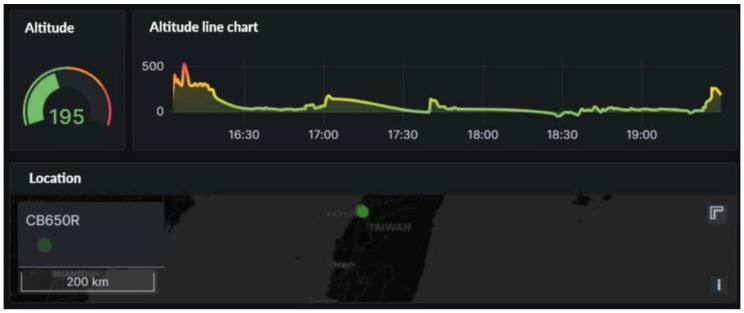
Vehicle coordinates.

In addition, this study provides a visual interface that combines altitude with the vehicle’s real-time location, allowing users to quickly locate the vehicle’s current location. By analyzing the relationship between altitude and vehicle position, users can gain a deeper understanding of the vehicle’s operating environment. The system facilitates real-time tracking via maps, thereby enhancing fleet managers’ and rental service providers’ real-time monitoring capabilities. Figure 17 points coordinates or locations of the vehicles.

### 4.3. Alarm and Notification Presentation

When abnormal situations occur, this study designed a multiple alarm system to immediately notify relevant contacts through the Line Notify service. Preset thresholds trigger these alarm systems, taking into account abnormal changes in key indicators like temperature, humidity, and speed, among others, to ensure prompt response during critical moments. The following Table 9 details the alarm threshold values of each indicator and the corresponding alarm mechanism.

**Figure 18:**
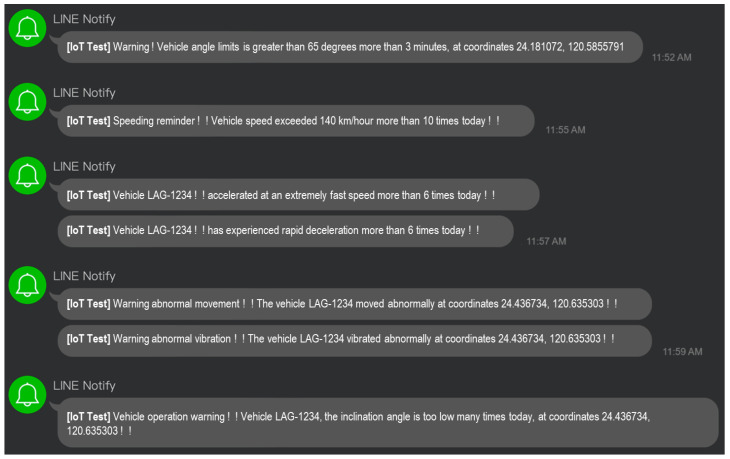
LINE notifications.

The example of LINE messenger notification is depicted in Figure 18 bellow.

### 4.4. Maintenance Testing

**Figure 19:**
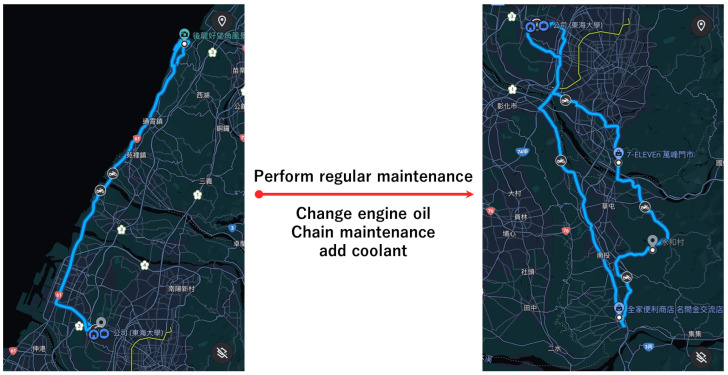
The route map of two rounds of maintenance testing.

This study implemented two rounds of comprehensive field tests as shown in Table 10 and Figure 19. With each round lasting more than an hour and covering diverse driving environments such as urban areas, mountainous areas, and highways, we aim to conduct an in-depth analysis of the long-term operational stability of the system. During the test, we performed a vehicle maintenance session, which included an oil change and coolant top-up, to assess the impact of routine maintenance on engine operating temperatures. To obtain a more comprehensive data analysis, we added monitoring of other key vehicle performance indicators and rigorously recorded all relevant data and activity details for subsequent analysis and evaluation.

**Figure 20:**
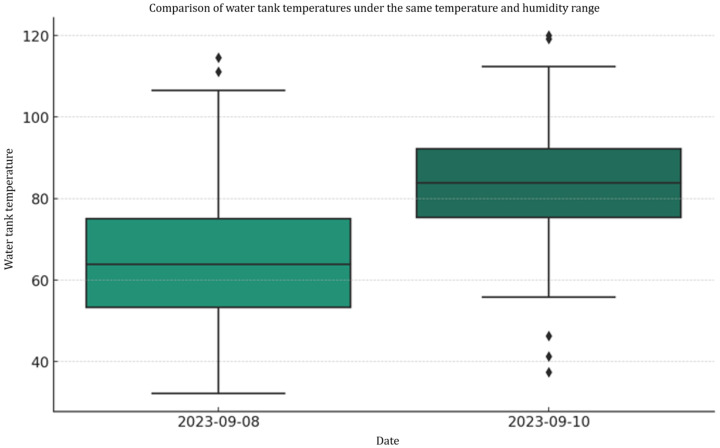
Comparison of water tank temperatures in the same temperature and humidity range.

In the two-day test, the ambient temperature and the water tank temperature showed a moderate positive correlation, and the correlation coefficients were 0.5820 and 0.5830, respectively. There is a moderate negative correlation between ambient humidity and water tank temperature, with correlation coefficients of −0.5518 and −0.4757. These data demonstrate how environmental factors affect engine operating temperatures. We selected data with the same ambient temperature and humidity range from the two experimental data sets. However, even after oil replacement and coolant addition maintenance, the engine operating temperature in the second experiment was still higher than in the first. This shows that although environmental conditions are impacted by engine temperature, maintenance operations are not the decisive factor in slowing down the temperature. Further observation revealed that the first experiment’s average vehicle speed was higher, potentially aiding the engine’s heat dissipation; however, the second experiment’s higher altitude may require higher speeds to climb the slope. We recommend a detailed study in a controlled environment in the future, as these variables may also influence engine temperature. Figure 20 describes the comparison of water tank temperatures under the same temperature and humidity range.

**Figure 21:**
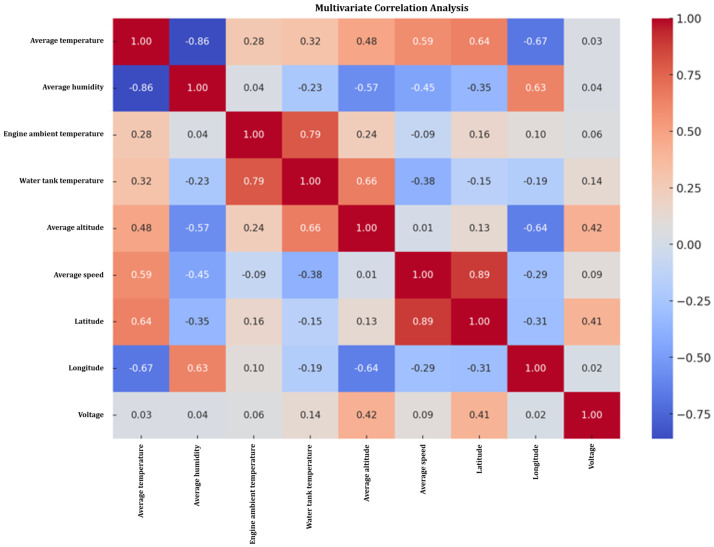
Multivariate analysis.

The multivariate correlation analysis in Figure 21 revealed a significant correlation between the water tank temperature and the ambient temperature and humidity, suggesting a potential increase trend in the water tank temperature under high temperature and low humidity conditions. In addition, there is a correlation between vehicle speed and engine ambient temperature, suggesting that vehicle speed may impact engine temperature. In comparison, the correlation between altitude and other variables is low, indicating that altitude may have a limited impact on these variables, but this finding still needs further empirical research to confirm.

### 4.5. Discussion

When deploying NB-IoT solutions, several challenges and limitations should be considered. One significant issue is network scalability, as connecting a vast number of devices can strain the current centralized server/client paradigm, potentially leading to bottlenecks in data transmission. Additionally, the integration of the NB-IoT with 5G systems presents challenges such as the lack of an agreed-upon end-to-end architecture, which complicates the development of a multi-layer, multi-application framework. Moreover, energy efficiency remains a critical concern, particularly because most NB-IoT devices are battery-operated and constrained in terms of processing and memory resources. This limitation can lead to increased energy stress on NB-IoT nodes, especially when compared to non-licensed band IoT networks like LoRa and Sigfox. Furthermore, the application’s reporting rate and the coverage class influence the lifetime of an NB-IoT device, making a balance between coverage and energy consumption essential. The performance of the NB-IoT can be affected in high-speed mobile conditions, as it is primarily optimized for static or low-speed scenarios, which may lead to challenges in maintaining stable connections and reliable data transmission.

The future direction for implementing the NB-IoT involves several key areas of focus aimed at enhancing its performance and applicability. They include defining testing parameters, such as the weather, the estimated temperature and humidity, and the wind speed, since these may influence the accuracy of the sensor while moving or not moving. One significant aspect is the development of novel techniques and approaches at all network layers, particularly in the context of energy efficiency, data rate performance, and scalability. It encourages researchers to explore the modeling of the physical layer of the NB-IoT network and propose energy-efficient techniques for modulation and coding scheme selection. Additionally, the integration of artificial intelligence and big data analysis technologies plays a crucial role in extracting valuable information from collected data, thereby improving the accuracy of anomaly detection and fault prediction. This integration will also provide intelligent decision support for management and enhance user experience through personalized system interactions. Moreover, the exploration of energy harvesting techniques, such as ambient backscatter, will further extend the operational lifetime of NB-IoT devices, thereby making them more sustainable in various applications. Overall, continuous technological innovation and application expansion will significantly contribute to the advancement of smart transportation systems and vehicle safety monitoring.

## 5. Conclusions

This research developed a real-time sport cruiser motorcycle monitoring system, based on the Internet of Things, integrating the NB-IoT, MQTT, database management, and data visualization technologies. This system not only promotes driving safety but also helps correct improper driving behavior. Data analysis shows that ambient temperature and humidity have a significant impact on engine temperature. Maintenance operations, such as oil changes and coolant refills, have a limited impact on engine temperature, and other factors, such as vehicle speed and altitude, must also be considered. This study not only proposes a practical real-time monitoring solution, it also serves as an important reference for future research on factors affecting engine operating temperature. Following a successful development of a real-time monitoring system for big motorcycles in this study, future prospects may focus on further technological innovation and application expansion. With the continuous advancement of the Internet of Things, NB-IoT technology, and sensor technology, exploring more efficient data transmission protocols and sensors for improving the data processing and transmission efficiency of the system will become an important research direction. At the same time, the system’s application to other vehicle types, such as electric motorcycles, and integration with urban traffic management systems are also worth exploring in the future. Furthermore, the use of artificial intelligence and big data analysis technology to extract valuable information from the collected data can improve the accuracy of anomaly detection and fault prediction, as well as provide intelligent decision support for fleet management. Future research will also focus on further optimizing the user experience and interactive design to make the system more friendly and personalized while strengthening user data security and privacy protection. We anticipate that continuous technological innovation and application expansion will significantly contribute to vehicle safety monitoring, management efficiency enhancement, and the development of smart transportation systems.

## Figures and Tables

**Table 1:** Alarm triggers.

Sensor	Alarm Triggers
GY-906	Temperature alarm: greater than 150 °C
JY-61	Reversing alarm: inclination angle is greater than 65° for more than 3 min
	Operation alarm: inclination angle is greater than 35°
GY-NEO6MV2	Location: Send vehicle coordinates
	Abnormal movement: Abnormal movement under flameout state
	Speed: 140 km/h over the speed limit
SW-420	Abnormal vibration: detect vibration when the engine is turned off
INA226	Low voltage: battery voltage is lower than 10 volts
	Voltage is too high: battery voltage is higher than 16 volts

**Table 2:** Specification of infrared temperature sensor.

Parameter Name	Details
Product name	GY-906
Working voltage	3.3–5 V
Working current	10 mA
Output interface	I2C
Measuring range	−40–125 °C
Accuracy	±0.5 °C
size	25 mm × 20 mm × 10 mm

**Table 3:** OLED screen specifications.

Parameter Name	Details
Working voltage	3.3 V or 5 V
Maximum current	20 mA
Pixel density	128 × 64
Communication interface	SPI, I2C
Screen size	1.3 inches

**Table 4:** Six-axis accelerometer specifications.

Parameter Category	Parameter Name	Condition	Details
Six-axis accelerometer	Measuring range		±16 g
	resolution	±16 g	0.0005 (g/LSB)
Six-axis gyroscope	Measuring range		±2000°/s
	resolution	±2000°/s	0.061 (°/s)/(LSB)
Tilt sensing	Measuring range	X: ±180° Y: ±90°	X: ±180° Y: ±90°
	Inclination accuracy		0.2°
	resolution	Place horizontally	0.0055°
Electrical characteristics	Supply voltage		5 V
	Working current		9 mA

**Table 5:** GPS sensor specifications.

Parameter Name	Details
Working voltage	3.3 V to 5 VDC
Communication interface	UART TTL, 9600 bps (default)
Working current	45 mA
Capture time	Cold start 27 s, hot start 1 s
Communication methods	NMEA, UBX Binary, RTCM
Horizontal positioning accuracy	2.5 m
Navigation update rate	1 Hz (maximum 5 Hz)
Size	36 × 26 mm
Weight	22 g

**Table 6:** NB-IoT specifications.

Parameter Name	Details
Support frequency band	B3/B5/B8/B18/B26
Communication transmission rate	Single-Tone: 25.5 kbps downlink, 16.7 kbps uplink
Multi-Tone: 25.5 kbps downlink, 62.5 kbps uplink	
communication protocol	UDP/TCP/CoAP/LwM2M/MQTT
Power supply characteristics	Low supply voltage range (2.1 V∼3.63 V)

**Table 7:** Specification of temperature and humidity sensor.

Parameter Name	Details
Measurement parameters	temperature, humidity
Working voltage	3.3–5 V DC
Real-time	Maximum 2.5 mA
Temperature range	−40 °C to 80 °C
Temperature accuracy	±0.5 °C
Humidity range	0–100% RH
Humidity accuracy	±2–5% RH
Size	15.1 × 25.4 × 7.7 mm

**Table 8:** Vibration sensor specifications.

Parameter Name	Details
Operating Voltage	3.3 V to 5 V DC
Operating Current	15 mA

**Table 9:** Sensor and notifications.

Sensor	Notification
GY-906	Temperature alarm: greater than 150 °C
YOU-61	Reversing alarm: the inclination angle is greater than 65° for more than 3 min Operation alarm: the inclination angle is greater than 35°
GY-NEO6MV2	Location: Send vehicle coordinates Abnormal movement: abnormal movement under flameout state Speed: 140 km/h over the speed limit
SW-420	Abnormal vibration: detect vibration when the engine is turned off
INA226	Low voltage: battery voltage is lower than 10 volts Voltage is too high: battery voltage is higher than 16 volts

**Table 10:** Driving information.

No.	Materials (pen)	Average Room Temperature (Degrees)	Average Altitude (m)	Average Speed (km/h)	Mileage of the Day (km)
1	192	30.30	83.00	70.99	75
2	247	29.79	150.37	36.78	109

## Data Availability

Data are contained within the article.
